# The Roles of Liver-Resident Lymphocytes in Liver Diseases

**DOI:** 10.3389/fimmu.2019.01582

**Published:** 2019-07-16

**Authors:** Yanan Wang, Cai Zhang

**Affiliations:** Institute of Immunopharmacology and Immunotherapy, School of Pharmaceutical Sciences, Shandong University, Jinan, China

**Keywords:** liver, residency, liver-resident lymphocytes, liver disease, immune-mediated disease

## Abstract

Tissue-resident lymphocytes usually reside in barrier sites and are involved in innate and adaptive immunity. In recent years, many studies have shown that multiple types of lymphocytes are resident in the liver, including memory CD8^+^ T (T_RM_) cells; “unconventional” T cells, such as invariant natural killer T (iNKT) cells, mucosal associated invariant T (MAIT) cells, and γδT cells; innate lymphoid cells (ILCs) such as natural killer (NK) cells and other ILCs. Although diverse types of tissue-resident lymphocytes share similar phenotypes, functional properties, and transcriptional regulation, the unique microenvironment of the liver can reshape their phenotypic and functional characteristics. Liver-resident lymphocytes serve as sentinels and perform immunosurveillance in response to infection and non-infectious insults, and are involved in the maintenance of liver homeostasis. Under the pathological conditions, distinct liver-resident lymphocytes exert protective or pathological effects in the process of various liver diseases. In this review, we highlight the unique properties of liver-resident lymphocytes, and discuss their functional characteristics in different liver diseases.

## Introduction

Lymphocytes are classically viewed as circulating immune cells that continuously traffick in blood, lymph nodes, and other secondary lymphoid tissues. Naïve T cells patrol the organs and are stimulated by antigen-presenting cells (APCs) that migrate from the site of infection to lymph nodes in infectious conditions. Then, T cells undergo clonal expansion and differentiation into effector cells that can migrate to the sites of inflammation. After the first infection, circulating memory T cells are established and are ready to mount a superior response to secondary infection (1, 2). However, recent studies have demonstrated the existence of tissue-resident lymphocytes (non-circulating lymphocytes) that usually reside in barrier sites (such as skin, lung, small intestine, and liver), but do not circulate into the periphery, and are involved in innate and adaptive immune responses (3–5). These specialized tissue-resident lymphocytes include memory CD8^+^ T (T_RM_) cells, unconventional T cells [e.g., natural killer T cells (NKTs), mucosal associated invariant T (MAIT) cells, γδT cells, CD8αα^+^ intraepithelial lymphocytes (IELs)], and innate lymphoid cells (ILCs).

Notably, many studies have revealed that the liver not only has metabolic activities, nutrient storage, and detoxification functions, but also is a complex immunological organ (6, 7). The liver has a unique characteristic morphologic organization, cell composition, and functions. Kupffer cells, the liver-resident macrophages, and DCs, comprise a significant number of the non-hepatocytes and act as a hepatic immune sentinel to remove or alert the immune system about the presence of harmful pathogens (8, 9). In terms of its lymphocyte composition, the lymphocytes in the liver are highly distinct from those in blood and lymphoid organs. Intrahepatic lymphocytes comprise enriched innate immune cells, such as NKT cells, ILCs, and γδT cells. NK and NKT cells constitute about 50% of total intrahepatic lymphocytes. When activated, NK and NKT cells play major roles in resistance to viral infection and regulation of innate and adaptive immune responses. Liver harbors all ILC subtypes including NK cells, ILC1, ILC2, and ILC3 (10). Different hepatic ILC subsets play important roles in protection against pathogen invasion, maintenance of tissue homeostasis, and repair of damaged tissue (10). Liver γδT cells account for 3–5% of the total liver lymphocytes and 15–25% of the total number of liver T cells, which is higher than that in peripheral blood (11). Therefore, the liver is regarded as an organ of predominant innate immunity (12, 13).

Recent studies have shown that many liver lymphocytes show tissue-resident feature. These cells include T_RM_ cells, ILCs (e.g., NK cells and other ILC subsets), γδT cells, and NKT cells (14–17). The newly identified liver-resident NK cells are regarded as hepatic ILC1s, based on their phenotypes (expressing high levels of CD49a and TRAIL, but lacking CD49b) (15, 18, 19). These CD49a^+^DX5^−^ liver-resident NK cells selectively reside in the liver sinusoids, and constitute about 50% of total liver NK cells (15, 20). These liver-resident lymphocytes serve as sentinels and frontline defenders in response to infection and non-infectious insults, and function in immunosurveillance, immune regulation, and maintenance of liver homeostasis. Exploring the phenotypes and functional characteristics of liver-resident lymphocytes would increase our understanding of hepatic immunity and lead to new or better treatments for liver diseases. In this review, we focus on the phenotypic and functional characteristics of liver-resident lymphocytes, their relationship with liver-related diseases, and potential therapeutic strategies.

## Hallmarks of Tissue-Resident Lymphocytes

Although tissue resident lymphocytes are diverse and locate in different tissues, they share many important characteristics in terms of their distribution, phenotypes, transcriptional regulation, and function. Tissue resident lymphocytes are abundant at barrier tissues (such as skin, lung, small intestine, and liver) where they recognize a wide variety of harmful signals, such as microbial products, infection, inflammation or tissue injury, and maintenance of tissue integrity. Upon sensing harmful or stress signaling, they produce antimicrobial and tissue-protective factors rapidly and they usually share a “memory-like” phenotype and function to provide long-lasting and robust protection against subsequent infection (21). Notably, long-term maintenance and rapid expansion of tissue-resident lymphocytes mainly depends on their local self-renewal ability (4, 22).

Tissue-resident lymphocytes share similar cell-surface phenotypes in accordance with their functional features and resident mechanisms. Many tissue-resident lymphocyte subsets express CD44, CD103, and CD49a, which mediate the adhesion and retention of these cells (23, 24). They usually lack the lymphoid homing markers, such as CCR7 and CD62L, and downregulate the expression of tissue egressing receptors, such as sphingosine-1-phosphate receptor 1 (S1PR1) and other members of S1P receptor family including S1PR4 and S1PR5 (25, 26). The S1P receptor mediates lymphocytes egress from tissue in a S1P gradient-dependent manner, while CD69 suppresses and internalizes the S1P receptor, thus inhibiting lymphocyte egress from lymphoid organs and promoting their retention (27, 28). In addition, chemokine receptors play an important role in the recruitment and homing of lymphocytes to specific organs. For example, CCR4 and CCR10 are highly expressed on skin-homing memory T cells (29). The migration or retention of lymphocytes in intestinal sites depend on the integrin α4β7 and CCR9 (30, 31). CXCR3, CXCR6, and CCR5 are involved in T-cell trafficking to the liver, as well as the maintenance of liver-resident cells (32–34).

Recent transcriptional analyses have demonstrated that tissue-resident cells display a unique pattern of transcription factor expression. BLIMP1 (B lymphocyte-induced maturation protein; encoded by *Prdm1*) and HOBIT (Homolog of BLIMP1 in T cells; encoded by *Znf683*) are involved in establishing diverse tissue-resident lymphocyte populations such as T_RM_, NKT, and NK cells (35–37). HOBIT and BLIMP1 downregulate *Ccr7, S1pr1*, and *klf2* (which encodes the Krüppel-Like Factor 2, a transcription factor targeting gene *S1pr1*), which are required for lymphocyte egress from tissues (25, 35). They also repress *Tcf7*, which encodes TCF1, a transcription factor required for the development of conventional circulating memory cells (26, 38). The T-box transcription factors, T-bet and EOMES are usually downregulated in T_RM_ cells in the skin, gut, lung, and brain, whereas, some level of T-bet expression is essential for IL-15-mediated T_RM_ survival (39–41). In addition, innate tissue-resident lymphocytes including iNKT cells and MAIT cells which have memory-like properties, have similar expression patterns of the transcription factor promyelocytic leukemia zinc finger (PLZF), which has been shown to be essential for the development and function of innate T cells (42–44).

## Phenotypic and Functional Characteristics of Liver-Resident Lymphocytes

Liver-resident lymphocytes share many common phenotypic and transcriptional characteristics with other tissue-resident lymphocytes, which are different from circulating lymphocytes. Conventional circulating lymphocytes usually express lymph node homing molecules CCR7 and CD62L and tissue egressing S1P receptors, and are regulated by transcriptional factors KLF2 and TCF1. Whereas, liver-resident lymphocytes express adhesion and retention molecules including CD103, CD69, CD49a, CD44, and chemokine receptors CXCR3 and CXCR6. BLIMP1 and HOBIT regulate the transcriptional programing of liver-resident lymphocytes. Functionally, circulating lymphocyte subsets play immune surveillance roles by patrolling blood, lymph nodes, and other secondary lymphoid organs, whereas the liver-resident lymphocytes reside in the liver sinusoidal blood, and focus on the hepatic homeostatic maintenance and immune defenses.

Although tissue-resident lymphocyte subsets share similarities in phenotype, function, and transcriptional regulation, different tissue microenvironments may reshape the tissue-resident lymphocytes, providing them with organ- or tissue-specific phenotypes and functions. For example, the phenotypic and functional characteristics of both mouse and human liver-resident NK cells have unique phenotypic and functional characteristics compared with other tissue-resident NK cells (Tables 1, 2). Although most tissue-resident NK cells are CD49a^+^DX5^−^, salivary gland-resident NK cells are CD49a and DX5 double positive (45, 46). Different organ-derived tissue-resident NK cells may have unique functions and play different roles in autoimmune diseases (19). The unique features of the hepatic lymphocytic composition and the unique immunological properties of the liver indicate that it is essential to clarify the unique properties of liver-resident lymphocytes and their roles in liver-related diseases.

**Table 1:** Phenotypic and functional characteristics of murine liver-resident NK cells and other tissue-resident NK cells.

	**Surface markers**	**Transcription factors**	**Effector molecules**
Liver	NK1.1^+^CD49a^+^DX5^−^CD69^+^ CD103^−^CXCR6^+^CD127^+/−^	T-bet^+^Eomes^lo^	IFN-γ^+^TNF-α^+^Granzyme^+^ GM-CSF^+^TRAIL^+^
Uterus	NK1.1^+^CD49a^+^DX5^+/−^CD69^+^ CD127^−^	T-bet^+^Eomes^+/−^	IFN-γ^+^TNF-α^+^Granzyme^+^
Salivary gland	NK1.1^+^CD49a^+^DX5^+/−^CD69^+^CD103^−^CD127^+/−^	T-bet^+^Eomes^+^	IFN-γ^+/−^TRAIL^+/−^
Adipose	NK1.1^+^CD49a^+^DX5^−^CD69^+^CD103^−^CD127^+/−^	T-bet^+^Eomes^−^	IFN-γ^+^TRAIL^−^
Kidney	NK1.1^+^CD49a^+^DX5^−^CD69^+^	ND	TRAIL^+/−^
Lung	NK1.1^+^CD49a^+^DX5^−^	ND	IFN-γ^+^ Granzyme^+^
Skin	NK1.1^+^CD49a^+^DX5^−^CD69^+^	Eomes^−^	ND

*ND, not determined*.

**Table 2:** Phenotypic and functional characteristics of human liver-resident NK cells and other tissue-resident NK cells.

		**Surface markers**	**Transcription factors**	**Effector molecules**
Liver	CD49a^+^	CD56^bright^CD49a^+^CD69^+^CD103^+^ CXCR6^−^	T-bet^hi^Eomes^lo/−^	IFN-γ^+^TNF-α^+^GM-CSF^+^ CD107a^+^Perforin^+^Granzyme^+^
	Eomes^hi^	CD56^bright^CD49a^−^ CD69^+^ CD103^−^ CXCR6^+^	T-bet^lo^Eomes^hi^	IFN-γ^+^TNF-α^+^GM-CSF^+^ CD107a^+^Perforin^lo^Granzyme^lo^
	CD49e^−^	CD56^bright^ CD69^+^ CXCR6^+^	T-bet^+^Eomes^hi^	IFN-γ^+^ TNF-α^+^ CD107a^+^
Uterus		CD56^bright^CD49a^+^CD103^+/−^ CD69^+/−^	Eomes^+^	IFN-γ^+^TNF-α^+^GM-CSF^+^ Perforin^+^ Granzyme^+^
Tonsil		CD56^bright^CD49a^+^CD69^+^ CD103^+^ CXCR6^+^integrinβ7^+^	T-bet^+^Eomes^+^	IFN-γ^+^CD107a^+^Perforin^+^ Granzyme^+^
Lung		CD56^bright^CD49a^+^CD69^+^ CD103^+^ CXCR3^+^	ND	IFN-γ^+^CD107a^+^ Granzyme^+^
Lymphoid tissue		CD56^bright^CD49a^−^ CD69^+^ CD103^−^ CXCR6^+^	Eomes^+^	IFN-γ^lo^CD107a^+^Perforin^+^ Granzyme^lo^

*ND, not determined*.

### CD8^+^ Liver-Resident Memory T Cells

Tissue-resident memory T cells (T_RM_) are a non-recirculating subset positioned in non-lymphoid tissues, such as skin, gut, lung, salivary glands, and the genital tract, which provide rapid and powerful responses to reinfection (47). Activated T_RM_ cells exert effector functions, including cytolytic activity and the secretion of proinflammatory cytokines such as IFN-γ and TNF-α. T_RM_ cells not only act as first line of defense by directly lysing target cells, but also confer protective functions through the recruitment of circulating T cells or other immune cells via chemokine production (48).

Studies have shown that an important subset of CD8^+^ T_RM_ cells resides in liver where they patrol the vasculature and provide protection against invading pathogens (49). A population of interleukin (IL)-2^hi^CD8^+^ T_RM_ cells expressing tissue retention signals resides in healthy human liver, and is expanded upon HBV infection (50). The high IL-2 production of these cells is likely to be critical to maintaining the antigen-specific proliferation and memory responses of hepatic CD8^+^ T_RM_ cells (51). CD8^+^ T_RM_ cells are present in the murine liver under conditions of Malaria infection (34, 52). CD8^+^ T_RM_ cells in human and murine livers share similar phenotypic features. CD69 and CD103 are two key markers expressed by T_RM_ cells in various non-lymphoid tissues. The majority of hepatic CD8^+^ T_RM_ cells express CD69; however, some of them lack expression of CD103 (35, 49, 52). Like other hepatic resident lymphocytes, hepatic T_RM_ cells express the liver homing chemokine receptors CXCR3 and CXCR6. Notably, CXCR6 is required for the maintenance of liver resident memory CD8^+^ T cells specific for infectious pathogens (34). Hepatic T_RM_ cells also express low levels of CCR7, CD62L, KLF2, and S1PR1, which are required for tissue exit (53, 54). Regarding transcriptional regulation, liver T_RM_ cells express HOBIT and BLIMP1, which can silence genes involving lymphocyte egress from tissues and repress the development of circulating memory cells (35). Recently, researchers found that the transcriptional repressor Capicua (CIC)–ETS translocation variant 5 (ETV5) axis is a key molecular module that regulates the expression of *Hobit* and controls liver CD8^+^ T_RM_ cell development to maintain normal liver function (55). Interestingly, using intra-vital imaging, researchers found that CD8^+^ T_RM_ cells patrol and reside in the hepatic sinusoids, which is dependent upon LFA-1–ICAM-1 interactions. Antigen-specific CD8^+^ T cells failed to form substantial liver resident memory populations following Plasmodium or lymphocytic choriomeningitis virus (LCMV) immunization in *Itgal*^−/−^ mice in which LFA-1 is deficient (56).

### Liver-Resident ILCs

ILCs are a family of innate immune lymphocytes lacking antigen-specific receptors, which mirror the phenotypes and functions of T cells. They are involved in the development of lymphoid tissue, tissue repair, and the maintenance of tissue integrity (57). Traditionally, ILCs are divided into three subsets: Group 1 ILCs (comprising conventional NK cells and ILC1s); Group 2 ILCs (ILC2s); and Group 3 ILCs [comprising ILC3s and lymphoid tissue inducer (LTi) cells] according to their cytokines expression, developmental pathways and functions (58). Recently, Vivier et al. proposed classifing ILCs into five subsets—NK cells, ILC1s, ILC2s, ILC3s, and LTi cells—based on their development and function especially the developmental trajectories (59). It is notable that NK cells are formerly classified as Group 1 ILCs, and now they are regarded as an independent subset of ILCs. ILCs usually localize at mucosal barriers. Long-term parabiosis experiments have identified that ILC subsets are resident in both lymphoid and non-lymphoid organs of adult mice. They are maintained by self-renewal during homeostasis and the acute infection environment (4). NK cells produce IFN-γ, perforin, and granzymes, and lyse tumor and virally infected cells. ILC1s are generally non-cytotoxic or weakly cytotoxic, and act as a first line of defense against viral and bacterial infections. ILC2s predominantly produce T helper 2 (T_H_2)-cell associated cytokines (including IL-4, IL-5, IL-9, and IL-13) and are involved in the innate immune response to parasites. They can also produce amphiregulin (AREG) to promote tissue repair. ILC3s produce T helper 17 (T_H_17)-cell associated cytokines (including IL-17A, IL-17F, and IL-22) and contribute to the control of extracellular microbes and maintain intestinal homeostasis. LTi cells produce lymphotoxin and are involved in the formation of secondary lymph nodes and Peyer's patches during embryonic development (58, 59). Recently, a regulatory subpopulation of ILCs (ILCregs) was identified in the gut that contribute to the resolution of innate intestinal inflammation (60).

A liver-resident subpopulation of murine NK cells was recently defined with a distinct CD49a^+^DX5^−^ phenotype, which is different from the conventional CD49a^−^DX5^+^ NK cells (15). These liver-resident NK cells differ from the conventional NK (cNK) cells in their phenotype, cytokine production, cytotoxicity, and developmental pathways. They express higher levels of CD69, CD44, CXCR3, and CXCR6, which are related to tissue residency (61), and CD160, compared with cNK cells. Compared with cNK cells in the liver and spleen, they do not express the lymphoid homing marker CD62L (62). Notably, CXCR6 is crucial for hepatic NK cell-mediated memory responses, suggesting that liver-resident NK cells may possess memory-like properties (63). Liver-resident NK cells produce higher levels of TNF-α and GM-CSF and similar amounts of IFN-γ, compared with liver and splenic cNK cells, which are key players in inflammatory responses. They express higher levels of TNF-related apoptosis-inducing ligand (TRAIL) and FasL, which indicate a higher cytotoxic capacity compared with cNK cells (61). TRAIL contributes to the NK-cell-mediated elimination of activated CD4^+^ T cells or virus-specific CD8^+^ T cells during chronic viral infection. TRAIL^+^ liver-resident NK cells may negatively regulate antiviral immunity in chronic viral infection, but also constrain viral-induced autoimmunity (64, 65). Bone marrow also contains a very small subpopulation of DX5^−^ NK cells which were considered to be immature NK cells. However, hepatic DX5^−^ NK cells expressed much higher CD11c and TRAIL compared with bone marrow DX5^−^ NK cells, indicating that liver-resident NK cells are phenotypically different from those of the bone marrow (15). Liver-resident NK cells also have different transcription factor requirements compared with cNK cells. They express lower levels of EOMES. The HOBIT-BLIMP1 transcriptional module is also required for tissue retention of liver-resident NK cells (35). They are generated from hepatic NK1.1^−^CD3^−^CD19^−^ hematopoietic progenitor cells (HPCs) and have a unique developmental pathway that does not require NFIL3, but depends on Tbx21 (15, 62, 66). The aryl hydrocarbon receptor (AHR) is required for the maintenance of liver-resident NK cells (67).

In humans, NK cells are divided into two main subsets, CD56^dim^ and CD56^bright^ NK cells, based on their expression of CD56 and CD16 (68). CD56^dim^ NK cells are the dominant population in the peripheral blood. In contrast, human liver is enriched in CD56^bright^ NK cells and most of them are described as liver-resident NK cells (36, 69, 70). Studies have shown that human liver-resident NK cells express retention-related molecules, such as CD69, CXCR6, and CCR5, while they lack expression of CD62L and CCR7, which are involved in the recruitment of circulating NK cells to lymphoid tissues (69, 70). HOBIT is involved in the regulation of tissue residency of human intrahepatic CD56^bright^ NK cells (36). Several different human liver-resident NK cell subsets have been found. It is gradually becoming accepted that there are two non-overlapping human liver-resident NK cell populations that are distinguish between human cNK and liver-resident cells by the expression of T-bet and EOMES (3, 71). EOMES^hi^T-bet^lo^ NK cells largely overlap with the CD56^bright^ and CXCR6^+^ NK cells that are located in the sinusoids, but are completely absent in blood (70, 72). In contrast, the EOMES^lo^T-bet^int^ phenotype is described for CD49a^+^ liver-resident NK cells, which are found in the parenchyma, although some EOMES^lo^ cNK cells recirculate freely (73). The function of the two human liver-resident NK cell subsets has not been clarified. The distribution and protein expression of the two subsets may provide clues to their different functions. EOMES^hi^ liver-resident NK cells are long-lived (they can persist for up to 13 years in sinusoids) and reside in the sinusoids and possibly recognize bacterial antigens coming from gut (71, 74). CD49a^+^ human liver-resident NK cells are found in the parenchyma and express cytotoxic effector molecules and receptors for MHC class I molecules, suggesting that they may perform immunosurveillance by recognizing and killing virally infected or cancerous hepatocytes. The expression of NKG2C suggested that CD49a^+^ human liver-resident NK cells may have memory-like properties (73). Interestingly, a human CD49e^−^ NK cell population identified by cytometry by time-of-flight (CyTOF) analysis was reported to be liver-resident. These CD49e^−^ liver-resident NK cells have similar capacities to produce cytokines and degranulate upon stimulation with PMA and ionomycin, although their exact function remain to be fully determined (75). Liver-resident NK cells are also reported to possess memory-like properties upon sensitization with haptens or virus-like particles (15, 21, 76). Liver-resident NK cells from influenza virus-infected mice conferred protective immunity against secondary influenza virus infection (21).

ILCs are abundant in the liver, with the dominant NK cells and ILC1s and the relatively rare ILC2s and ILC3s (77). Several studies have shown that hepatic ILC1s and ILC2s have features of liver residence (15, 78, 79). Based on the phenotypes of CD49a^+^ TRAIL^+^ CD49b^−^, the liver-resident NK cells were regarded as hepatic ILC1s (15). Human intrahepatic ILC2s express the tissue residence marker CD69. The majority of liver-derived ILC2s express chemokine receptor CCR6, allowing them to respond to CCL20 secreted by the biliary epithelium. They express high levels of integrins, such as Very Late Antigen-5 (VLA-5) and VLA-6, which bind fibronectin and laminin in normal and inflamed livers (79). ILC1s and ILC2s are both developed from common helper innate lymphoid progenitors (CHILPs) and innate lymphoid cell precursors (ILCP), depending on the transcription factor PLZF (59).

### Liver-Resident γδT Cells

γδT cells, a subset of innate-like T lymphocytes, are enriched in the mucosal surface and are defined as tissue-resident lymphocytes in skin, lung, and intestinal mucosa, where they serve as sentinels and exert immunosurveillance functions in response to infection and tumorigenesis (80–85). γδT cells are also abundant in liver at a frequency of 3–5% of all intrahepatic lymphocytes and predominantly IL-17-producing γδT cells. A mouse parabiosis model demonstrated the residency of hepatic γδT cells (16). Lipid antigens from gut commensal microbes presented by hepatocyte-expressed CD1d can be recognized by the γδTCRs (T-cell receptors) of liver-resident γδT cells, which supports their development and homeostasis (16). Murine CD8αα γδT cells, but not CD8αβ γδT cells, are recently identified as liver resident. A class Ib MHC molecule H2-Q10, which is highly expressed in liver, is confirmed as a new high affinity ligand for CD8αα and controls the development of liver-resident CD8αα γδT cells (86). In humans, a recent study found that a CD27^lo^CD45RA^lo^ subset of Vδ1^+^ T cells expressing enhanced levels of liver-resident associated marker CD69, CXCR3, and CXCR6 reside in the liver. This population is functionally distinct from equivalent subsets in peripheral blood. They produce significantly more pro-inflammatory cytokines IFN-γ and TNF-α and may play roles in chronic liver disease, such as CMV infection (87). More recently, several lines of evidence have shown that γδT cells form long-lived memory populations upon local inflammation or infection (88, 89). Tissue-resident γδT cells have been implicated in the protection processes such as sustaining tissue homeostasis and control pathogen infection, but their presence may also exacerbate local inflammation under certain circumstances (90). However, the exact phenotypic or functional characteristics of liver-resident γδT cells require further study.

### Hepatic NKT Cells

NKT cells are innate-like T cells that express TCR and recognize both exogenous and endogenous lipid antigens presented by a class I MHC-like molecule, CD1d. Based on the differences in TCR usage and the various antigens that they recognize, NKT cells can be divided into two main subsets, type I or invariant NKTs (iNKTs), expressing an invariant TCR recognizing α-galactosylceramide (α-GalCer), and type II NKT cells, displaying a diverse TCR repertoire that can recognize sulfatide or other glycolipids (91, 92). Following activation, type I NKT cells can secrete different kinds of cytokines and further stimulate dendritic cells (DCs), NK cells, B cells, and T cells, thus playing an important immunoregulatory role in both innate and adaptive immune responses. However, the activation of type II NKT cells by sulfatide does not induce the activation of B, NK, or T cells. Accumulating evidence has shown that the two NKT subsets may vary in their roles in health and disease. Type I NKT cells predominantly play pro-inflammatory roles in inflammatory conditions, while type II NKT cells play anti-inflammatory or immunosuppressive roles in several experimental models (93, 94).

NKT cells are generally known as tissue-resident lymphocytes. Similar to other liver-resident lymphocytes, NKT cells are mostly positive for CD49a and CD69. Both human and mouse NKT cells express high levels of CXCR6. The CXCR6–CXCL16 interaction not only mediates the accumulation, maturation, and survival of recent thymic emigrants in the liver, but also regulates the activation of NKT cells (95–97). Hepatic NKT cells express high levels of the adhesion molecule LFA-1, and LFA-1–ICAM-1 interactions result in long-term residence of NKT cells in the liver. Notably, PLZF is an NKT cell-specific transcription factor. PLZF orchestrates a part of innate-like phenotype of NKT cells via a set of target genes such as *Id2* (encoding ID2), *Maf* (encoding c-Maf), *Icos* (encoding c-ICOS), *Il12rb1*, and *Il18r1* that are essential for cytokine secretion, survival, co-stimulation, and responsiveness to proinflammatory cytokines (98). PLZF plays essential roles in the accumulation of hepatic NKT cells by up-regulating LFA-1 and down-regulating CD62L and KLF2 (43, 99). Transcriptional regulator Inhibitor of DNA-binding-2 (ID2) controls the survival of hepatic NKT cells by regulating the expression of CXCR6 and anti-apoptotic molecules Bcl-2 and Bcl-X_L_ (100). HOBIT-BLIMP1 transcriptional programing is also required for the maintenance of liver NKT cells and they function by suppressing lymphocyte egress genes, such as *Klf2, S1pr1, Tcf7*, and *Ccr7*. Numbers of NKT cells lacking both HOBIT and BLIMP1 were reduced in the liver (35).

## Roles of Liver-Resident Lymphocytes in Liver Diseases

### Liver-Resident Lymphocytes in Viral Infection of Liver

CD8^+^T_RM_ cells are believed to exert stronger anti-viral immune responses than circulating memory T cells do. The role of CD8^+^T_RM_ cells in the protection and control of hepatotropic viral infection has been demonstrated. Preliminary experiments demonstrated an essential role of liver-resident CD8^+^T_RM_ cells in long-term protection from chronic hepatitis C. Virus-specific CD8^+^ T_RM_ cells can reside in the liver for months or even years after primary viral infection. A second infection by HCV can be controlled rapidly, partly because of the rapid acquisition of the virus-specific cytolytic activity of hepatic CD8^+^T_RM_ cells (101). Researchers also found a distinct population of CD8^+^T_RM_ cells that are enriched in the liver of patients with chronic HBV infection, and observed an inverse correlation between T_RM_ frequency and HBV titer. T_RM_ cells express high levels of perforin, which may contribute to the direct killing of infected hepatocytes. Notably, T_RM_ cells express high-levels of cell-autonomous IL-2 that allows them to survive and overcome PD-L1-mediated inhibition to maintain immediate noncytolytic antiviral functions (50, 102). Moreover, Chun et al. found that CD8^+^ T_RM_ cells were enriched in HBV-related HCC compared with non-viral-related HCC and indicated a good prognosis (103). All of above results suggested that T_RM_ cells could contribute to a functional cure of hepatic viral infection and viral-related HCC. Therapeutic expansion of virus-specific T_RM_ cells might effectively control viral infection and related tumors.

The exact roles of liver-resident NK cells in hepatic viral infection remain poorly understood. The high expression of TRAIL and the efficient production of multiple cytokines, such as IFN-γ, TNF-α, and granulocyte-macrophage colony-stimulating factor (GM-CSF), indicate the potential importance of liver-resident NK cells in viral clearance. Human liver-resident EOMES^hi^ T-bet^lo^ CD56^bright^ NK cells display higher expression of activating receptors, such as NKG2D, NKp44, and NKp46, which recognize stress-induced ligands and viral-associated antigens. They also express a higher level of CD107a and perforin to provide a potent cytotoxic cellular response and enhanced degranulation (70). These properties suggest that liver-resident NK cells may spontaneously lyse virus-infected hepatocytes and promote Th1 polarization via secreting IFN-γ, thereby contributing to viral clearance in the liver. However, because of their high expression of immunosuppressive molecules, such as NKG2A, LAG-3 and CD39, liver-resident NK cells may exert negative a regulatory role that would contribute to the maintenance of liver immunotolerance (15, 78, 104, 105). Upon hepatic viral infection, NKG2A signaling in liver-resident NK cells inhibits the CXCL9 expression required for the infiltration of peripheral CD49b^+^ cNK cells into the liver. Blocking or creating a deficiency of NKG2A in liver-resident NK cells increased the numbers of IFN-γ-producing CD49b^+^ cNK cells, which further activated liver CD103^+^ DCs, leading to enhanced antigen-specific, anti-viral CD8^+^ T cell responses required for the clearance of hepatic viral infections (78). Therefore, inhibition of NKG2A signaling in liver-resident NK cells may be a novel vaccine strategy to improve CD8^+^ T cell responses against persistent liver infections. Interestingly, liver-resident NK cells negatively regulated the antiviral activity of hepatic T Cells via the PD-1–PD-L1 interaction during acute and chronic LCMV infection and adenovirus infection. The number and cytokine-producing levels of virus-specific T cells increased, accompanied by reduced viral loads in liver-resident NK-cell-deficient mice, while transferring of liver-resident NK cells into liver-resident NK-cell-deficient or wild-type mice suppressed hepatic T cell function during viral infection. The inhibitory effect of liver-resident NK cells on T cells can be abrogated by blockade of PD-L1 (105).

Human intrahepatic Vδ2^−^ γδT cells were highly clonally focused, among which the CD45RA^lo^CD27^lo^Vδ1^+^γδT cells were proved to be liver-resident. These hepatic Vδ1^+^ γδT cells are competent producers of IFN-γ and TNF-α (87). CMV infection is one of the drivers of hepatic Vδ2^−^ γδT cell infiltration, expansion, and memory formation. It is hypothesized that liver-resident or memory γδT cells have a protective effect against infection or tumor formation, and thus exert a critical role in local hepatic immunosurveillance (90). The exact characteristics of liver-resident or memory γδT cells and their roles in liver-related diseases require further investigation.

Type I NKT cells play a major role in controlling HBV or HCV infections via IFN-γ secretion, which inhibits HBV/HCV replication and stimulates adaptive immune responses, particularly in the early stages of infection (106, 107). However, NKT cells may also contribute to liver injury during chronic viral hepatitis infection by secreting of pro-inflammatory cytokines and inducing hepatocyte apoptosis (108).

### Liver-Resident Lymphocytes in Parasite Infection of Liver

Liver-resident T_RM_ cells are necessary to resist anti-parasite infection. A study has demonstrated that resident memory T lymphocytes can be induced during *Leishmania infantum* infection in the liver and may play a protective role (109). In addition, CD8^+^T_RM_ cells patrol the liver sinusoids and form the frontline defense against Malaria liver-stage infection (34, 52, 54). A prime-and-trap strategy that first activates T cells in the spleen and then traps them in the liver efficiently induces liver T_RM_ cells and protects against sporozoite challenge. At the first stage, anti-CLEC9A antibodies were used to target malaria antigens to CD8α^+^DCs to efficiently prime malaria-specific CD8^+^ T cells. At the next stage, the liver was infected with adeno-associated virus (AAV) expressing the malaria antigen to trap circulating primed CD8^+^ T cells in the liver and then drive T_RM_ cell formation to protect against Malaria infection (52). Recently, researchers created a heterologous “prime-and-trap” regimen, combining CD8^+^ T cell priming by gene gun-administered DNA vaccines and boosting with liver-homing radiation-attenuated sporozoites to induce high-frequency liver T_RM_ cells and achieved complete protection against sporozoites challenge (110). The efficacy of liver-resident T cells can be enhanced by intravenous administration of a malaria vaccine, resulting in expansion of pathogen-specific CD8^+^ T cells to provide long-term protection against malaria (111). Although there are various challenges, it will be significant to extrapolate these murine findings of Plasmodium-specific liver-resident CD8^+^T_RM_ cells to human malaria research and generate malaria vaccine for human in the future (112).

Up to now, there is rarely evidence about the role of other liver-resident lymphocytes in parasite infection of liver.

### Liver-Resident Lymphocytes in Hepatic Inflammatory Diseases

Liver-resident lymphocytes are involved in the pathogenesis of hepatic inflammatory-related diseases, such as hepatitis, liver fibrosis, liver cirrhosis, and non-alcoholic fatty liver disease (NAFLD).

Liver-resident NK cells play an important role in inhibiting or limiting liver fibrosis by killing activated hepatic stellate cells (19, 113). However, another study reported a high frequency of CD49a^+^ liver-resident NK cells that express CD25, CD34, and CXCR3 in cirrhotic livers. These CD49a^+^CD25^+^ liver-resident NK cells exhibited a high proliferative capacity in response to low doses of IL-2, and thus might contribute to liver inflammation and fibrosis (114). A DX5^−^CD11c^hi^ liver-resident NK cell subset was recently found to play an immunosuppressive role in autoimmune cholangitis by inhibiting CD4^+^ T cell proliferation and increasing the expression of genes involved in negative regulation of immune response in the inflammatory microenvironment (115).

ILC2s had a pro-inflammatory effect in a murine model of Con A-induced hepatitis. Hepatic ILC2s were activated and expanded via CD4 ^+^ T cell-mediated tissue damage and elevated IL-33, and then secreted IL-13 and IL-5, which led to further accumulation of eosinophils in the liver, thus aggravating liver tissue damage (116). Liver-resident ILC2s have pro-fibrotic effects in hepatic fibrosis. Under chronic hepatocellular stress, ILC2s accumulated and activated in the liver via ST2-dependent signaling resulting from elevated IL-33 (117, 118). ILC2s produce IL-13, which in turn triggers hepatic stellate cells (HSCs) activation in an IL-13Rα1- and STAT6-dependent fashion, and then aggravate hepatic fibrosis (119). A study on patients with inflammatory liver diseases demonstrated that the proportion of liver-resident ILC2s correlated positively with worsening liver function. ILC2s may contribute to ongoing fibrogenesis in liver disease through IL-13 and amphiregulin (79). However, there were reports showing that liver-resident ILC2s may have a protective response to repair tissue damage. In an study using an adenovirus (Ad)-induced liver hepatitis model, ILC2s were expanded via the increased expression of IL-33 and its receptor ST2, and then attenuated T cell-mediated liver injury by inhibiting TNF-α production (120). Another research found that IL-33–ST2 signaling protects against Con A-induced hepatitis by preventing Th1 and Th17 cell-mediated hepatic immune responses (121).

Hepatic ILC3s are involved in protection or pathogenesis via secretion of IL-22 in some liver diseases. ILC3s produce IL-22 and protect against liver injury induced by carbon tetrachloride (CCL4), Con A, and alcohol by activating STAT3 signaling (122). In contrast, in a study of HBV-infected patients and HBV transgenic mice, IL-22 played a pathological role in exacerbating chronic liver inflammation and fibrosis by recruiting hepatic Th17 cells (123). Recently, researchers demonstrated the pro-fibrotic role of ILC3s in both human and mouse liver fibrosis progression. ILC3s not only directly promote LX-2 (a human hepatic stellate cell line) fibrogenesis by producing IL-17A and IL-22 but also produce IL-22 to suppress IFN-γ production by other immune cells to exert indirect fibrogenic effects (124). More studies are required to clarify the respective roles of ILCs in different liver diseases and in different stages of disease progression.

γδT cells are a direct and potent source of critical inflammatory cytokines such as IFN-γ, TNF-α, and IL-17A in many pathological process. There is some evidence that liver-resident γδT cells are involved in the progression of NAFLD. Obesity-driven activation of the IL-17 axis is central to the development and progression of NAFLD (125). Increased numbers of γδT17 cells were found in the livers of high-fat diet (HFD)-fed mice and their NAFLD symptoms were reduced in *Tcr*δ^−/−^ mice, suggesting that hepatic resident γδT17 cells are one of the main sources of IL-17A in the liver during NAFLD and can accelerate NAFLD progression (16). The microbiota functions as a co-factor to accelerate HFD/high fat-high carbohydrate diet (HFHCD)-triggered NAFLD via increasing the number of liver-resident γδT17 cells.

Type I NKT cells usually play pro-inflammatory role and promote liver injury in the majority of chronic liver diseases, including hepatic ischemia reperfusion injury (IRI), Con A-induced hepatitis, primary biliary cirrhosis (PBC), and NAFLD (92, 126, 127). The activated type I NKT cells secrete large amounts of pro-inflammatory cytokines, which further recruit the accumulation of myeloid cells and neutrophils, and promote the activation of HSCs and NK cells, resulting in steatosis, fibrosis, hepatocyte necrosis, and even the development of HCC (92). Hepatic type I NKT cells also accumulate and activate, which are mediated by Kupffer cell-derived NLRP3 inflammasome activation and IL-1β release, subsequently promoting liver inflammation, neutrophil infiltration, inducing alcoholic liver injury in models of alcoholic liver disease (ALD), and exacerbating the disease progression (128). Activated hepatic type I NKT cells inhibit liver regeneration by producing high levels of IFN-γ in inflammatory conditions such as after partial hepatectomy (129). Type I NKT cells may also play a protective role in some conditions. The regulatory activity of NKT cells has been described in mice and mediates immune suppression through interaction with myeloid derived suppressor cells (MDSCs) or the production of IL-10 (130, 131). A recent report demonstrated that type I NKT cells could acquire a regulatory function and suppress T effector lymphocytes upon culture with rapamycin or TGF-β (132). However, whether hepatic type I NKT cells have regulatory or protective effects in liver inflammatory diseases has not been reported.

In contrast to the predominantly pro-inflammatory role of type I NKT cells, type II NKT cells suppress the pro-inflammatory response induced by type I NKT cells and consequently protect against liver damage. Activated type II NKT cells promote the recruitment of type I NKT cells into mouse livers; however, the interaction between type II and type I NKT cells leads to the tolerization of cDCs and anergy of type I NKT cells, thus further inhibiting adaptive immunity and suppressing neutrophil recruitment into the liver, which attenuates Con A-induced hepatitis (133, 134). The opposing roles of type I and type II NKT cells have also been demonstrated in IRI, ALD, NAFLD, parasite infection, and autoimmune diseases (92, 94, 134).

### Liver-Resident Lymphocytes in Hepatocellular Carcinoma

The liver is one of the most common sites for cancer in the body. It is a frequent target of both primary and secondary tumors. Although CD8^+^T cells are important effector cells to killing tumor cells, CD8^+^T_RM_ cells in anti-tumor immune responses has yet to be fully clarified. CD103^+^T_RM_ cells have been found to accumulate in tumor sites in several human solid tumors, including HCC (135–137). The abundance of CD8^+^T_RM_ cells was shown associated with prolonged survival and better prognosis in patients with HCC (103).

Liver-resident NK cells are enriched in HCC. The high expression of TRAIL, CD107a and perforin, as well as the efficient production of multiple cytokines, such as IFN-γ, TNF-α, suggest the potential importance of liver-resident NK cells in tumor control. It is reported that up to 79% of intratumoral NK cells had the CXCR6^+^CD69^+^ liver-resident phenotype. However, the tumor microenvironment impaired the antitumor function of liver-resident NK cells displayed by down-regulation of NKG2D and reduced capacity for cytotoxicity and production of cytokines (138). Importantly, IL-15 can recover the impaired anti-tumor function of liver-resident NK cells (138). A recent study reported a significant depletion of liver-resident NK cells from tumors of colorectal liver metastasis. This depletion of liver-resident NK cells correlated with hepatic recurrence post-resection. They further demonstrated that the accumulation of lactate in the tumor microenvironment caused a reduction in intracellular pH in hepatic NK cells, leading to mitochondrial dysfunction and apoptosis of liver-resident NK cells (139). This finding highlights the immunosurveillance role of liver-resident NK cells against tumor, and provides a promising therapeutic approach to restoring local NK-cell activity.

NKT cells play an important role in anti-tumor immunity. Hepatic NKT cells that are activated by α-GalCer administration or stimulated by HCC-derived antigens *ex vivo* contribute to suppressing the growth of hepatocellular carcinoma and eliminating disseminated hepatoma cells in the murine liver (92, 140). Recently, a study reported a relationship between gut microbiome-controlled bile acid metabolism and hepatic NKT cell-mediated antitumor immunosurveillance. In both primary and metastatic liver tumor models, depleting gut commensal bacteria significantly enhanced the accumulation of CXCR6^+^ NKT cells into the liver via a bile acid/CXCL16/CXCR6 axis, and further induced a liver-selective antitumor effect. The accumulated hepatic CXCR6^+^ NKT cells activated and produced more IFN-γ upon antigen stimulation, and inhibit the tumor growth in the liver (141). These findings imply good prospects for NKT cells in future immunotherapy for HCC and other cancers.

## Conclusion and Perspectives

**Figure 1:**
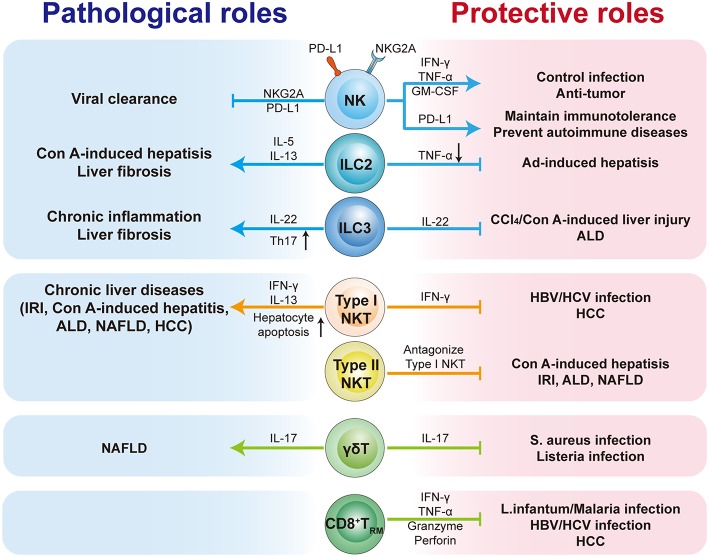
The pathological or protective roles of liver-resident lymphocytes in liver diseases. The liver contains multiple types of tissue-resident lymphocytes, including CD8^+^T_RM_ cells, NK cells, ILCs, γδT cells, and NKT cells. Liver-resident lymphocytes serve as the first line of defense in response to infection and non-infectious insults, and play roles in immunosurveillance, immune regulation, and the maintenance of liver homeostasis. Liver-resident lymphocytes generally exert protective or pathological effects via producing antimicrobial or homeostatic effector molecules, and by cooperating with other immune cells. They are involved in many kinds of liver diseases, such as metabolic liver diseases (e.g., ALD and NFALD), acute liver injury, liver fibrosis, viral hepatitis, and hepatic carcinoma (HCC). Modulation of liver-resident lymphocytes responses may represent promising therapeutic method to treat liver diseases.

As an immunologically complex organ, the liver contains multiple types of tissue-resident lymphocytes. Intense research over recent decades has described the phenotypic and transcriptional characteristics of liver-resident lymphocytes. They share many hallmarks with other tissue-resident lymphocytes and integrate signals within the hepatic microenvironment to produce certain unique features. Through residing and patrolling in the liver, they serve as sentinels and perform immunosurveillance in response to infection and non-infectious insults, and are critical in immune regulation and the maintenance of liver homeostasis. Under pathological conditions, distinct liver resident subsets are uniquely involved in the process of various liver diseases, exerting protective or pathological effects (Figure 1; Table 3). A deeper and more comprehensive understanding of liver-resident lymphocytes and their functional characteristics; and the cellular and molecular interactions among different liver-resident subsets, and other innate and adaptive lymphocytes, and the liver microenvironment will be vital to develop novel therapeutic strategies for diverse liver diseases.

**Table 3:** The role of liver-resident lymphocytes in liver diseases.

**Protective response**	**Liver diseases**	**Pathological response**
**CD8**^**+**^**T**_**RM**_ (Producing IFN-γ, TNF-α, Granzyme, and Perforin)	**Viral infection** (HBV, HCV, LCMV infection)	**Liver-resident NK** (Expressing NKG2A and PD-L1 to suppress T cell function)
**Liver-resident NK** (Producing IFN-γ, TNF-α, and GM-CSF)		
**Liver-resident type I NKT** (Producing IFN-γ)		
**CD8**^**+**^**T**_**RM**_ (Producing IFN-γ and TNF-α)	**Parasite infection** (*L.infantum* infection, Malaria infection)	
**Liver-resident NK** (Killing activated HSCs)	**Hepatic inflammatory diseases** (Hepatitis, Liver fibrosis, Liver cirrhosis, NAFLD, ALD)	**Liver-resident ILC2s** (Producing IL-5 and IL-13)
**Liver-resident ILC2s** (Inhibiting TNF-α production)		**Liver-resident ILC3s** (Producing IL-22 to recruit Th17 cells)
**Liver-resident ILC3s** (Producing IL-22)		**Liver-resident** **γ*δ*T** (Producing IL-17)
**Liver-resident type II NKT** (Antagonizing type I NKT)		**Liver-resident type I NKT** (Producing IFN-γ and IL-13)
**CD8**^**+**^**T**_**RM**_ (Producing IFN-γ and TNF-α)**Liver-resident NK** (Producing IFN-γ and Granzyme)	**Hepatocellular carcinoma**	
**Liver-resident type I NKT** (Producing IFN-γ)		

## Author Contributions

All authors listed have made substantial, direct, and intellectual contribution to the work and approved it for publication.

### Conflict of Interest Statement

The authors declare that the research was conducted in the absence of any commercial or financial relationships that could be construed as a potential conflict of interest.
